# The hypoxia-inducible factor 1 pathway plays a critical role in the development of breast muscle myopathies in broiler chickens: a comprehensive review

**DOI:** 10.3389/fphys.2023.1260987

**Published:** 2023-08-31

**Authors:** Nabeel Alnahhas, Eric Pouliot, Linda Saucier

**Affiliations:** ^1^ Department of Animal Science, Faculty of Agricultural and Food Sciences, Université Laval, Quebec, QC, Canada; ^2^ Olymel S.E.C./L.P, Boucherville, QC, Canada; ^3^ Institute of Nutrition and Functional Foods, Université Laval, Quebec, QC, Canada; ^4^ Swine and Poultry Infectious Diseases Research Center, Université de Montréal, Saint-Hyacinthe, QC, Canada

**Keywords:** wooden breast, white striping, spaghetti meat, hypoxia, HIF-1, broiler chickens

## Abstract

In light of the increased worldwide demand for poultry meat, genetic selection efforts have intensified to produce broiler strains that grow at a higher rate, have greater breast meat yield (BMY), and convert feed to meat more efficiently. The increased selection pressure for these traits, BMY in particular, has produced multiple breast meat quality defects collectively known as breast muscle myopathies (BMM). Hypoxia has been proposed as one of the major mechanisms triggering the onset and occurrence of these myopathies. In this review, the relevant literature on the causes and consequences of hypoxia in broiler breast muscles is reviewed and discussed, with a special focus on the hypoxia-inducible factor 1 (HIF-1) pathway. Muscle fiber hypertrophy induced by selective breeding for greater BMY reduces the space available in the *perimysium* and *endomysium* for blood vessels and capillaries. The hypoxic state that results from the lack of circulation in muscle tissue activates the HIF-1 pathway. This pathway alters energy metabolism by promoting anaerobic glycolysis, suppressing the tricarboxylic acid cycle and damaging mitochondrial function. These changes lead to oxidative stress that further exacerbate the progression of BMM. In addition, activating the HIF-1 pathway promotes fatty acid synthesis, lipogenesis, and lipid accumulation in myopathic muscle tissue, and interacts with profibrotic growth factors leading to increased deposition of matrix proteins in muscle tissue. By promoting lipidosis and fibrosis, the HIF-1 pathway contributes to the development of the distinctive phenotypes of BMM, including white striations in white striping–affected muscles and the increased hardness of wooden breast–affected muscles.

## 1 Introduction

Poultry meat is increasingly popular, and is increasingly consumed worldwide. According to the OECD (2022), meat consumption has been shifting toward poultry meat, and this trend is expected to continue in the long term. The popularity of poultry meat can be explained in high-income countries by consumer preference for white meat that is seen as a healthier choice than red meat. In low- and middle-income countries, this trend is mainly driven by the lower price of poultry meat than for other meat-producing animal species (OECD, 2022). In addition, poultry meat production and consumption are not subjected to traditional or religious restrictions.

In response to this increased popularity and to the growing world population, poultry meat production has been constantly increasing since the 1960s. In 2020, poultry meat accounted for 32% of worldwide meat production, slightly behind pork (36%) and well above meat produced from other species. Poultry meat production is projected to increase by 16% and to account for nearly half of all meat consumed between 2020 and 2031 (OECD, 2022).

The ability of the poultry industry to meet this increasing demand is based on its capacity to develop broiler strains with improved growth rates, higher feed efficiencies, and greater meat yield. This is achieved mainly by genetic selection, improved management, and optimized nutritional strategies. Early studies comparing strains of broiler chickens over time showed that genetic selection accounted for 80%–90% of achieved progress in broiler performances, while improved nutritional and feeding strategies accounted only for 10%–20% of this progress (Havenstein et al., 2003a; Havenstein et al., 2003b). In a more recent study, Zuidhof et al. (2014) compared broiler strains from 1957, 1978, and 2005 and showed that broiler growth rate had increased by 400% between 1957 and 2005 with a 50% reduction in feed conversion ratio. This study also showed an increase of 79% and 85% in the yield of the *Pectoralis major* muscle over this same period in male and female broilers, respectively. In modern commercial broiler chickens, such as Ross 308 weighing 2.6 kg, breast meat accounts for 25.1% and 27.2% of body weight in male and female broilers, respectively.

As it will be discussed in the following sections, the increased development of the pectoral muscles in commercial strains of broiler chickens has not been without consequences for the quality of broiler breast meat. In fact, multiple breast meat quality defects have emerged in recent years including breast muscle myopathies (BMM) of non-infectious origin such as white striping (WS), wooden breast (WB), and spaghetti meat (SM). These myopathies, which are moderately to highly (50%–65%) determined by genetics (Alnahhas et al., 2016; Lake et al., 2021), are associated with significant negative economic consequences for the poultry industry (Kuttappan et al., 2016; Barbut, 2019), and research is underway to develop strategies to reduce their occurrence and severity.

This review will summarize the available literature on the role of hypoxia in the development of BMM and will specifically elucidate the relationship between the hypoxia-inducible factor 1 (HIF-1) pathway and the development of the pathological changes associated with BMM. In Section 2, a brief overview of the characteristics of the *P*. *major* muscle is presented. In Section 3, the major structural and biochemical changes associated with selection for increased BMY are briefly discussed. The histopathological and biochemical characteristics of myopathic muscles are briefly described in Section 4. Sections 5 and 6 discuss the role of the HIF-1 pathway in the development of the histopathological and biochemical characteristics observed in myopathic muscles. The role of hypoxia in inducing apoptosis in myopathic muscles is discussed in Section 7. A summary of the most probable cascade of events between the activation of the HIF-1 pathway and the development of the phenotypic characteristics of BMM is presented in Section 8. Finally, conclusions and research perspectives are presented in Section 9.

## 2 A brief overview of the pectoral muscles in broiler chickens

The pectoral muscles in avian species are skeletal muscles that are responsible for producing the mechanical force required for flight. This section will focus on the *P*. *major* muscle, the target of breast muscles myopathies. The primary role of this muscle is to produce the force necessary for wing downstrokes, in contrast to the *P. minor* muscle, which powers wing upstrokes (Cao and Jin, 2020).

In terms of structure, the *P. major* muscle, similar to other skeletal muscles, is composed of striated muscle fibers. These fibers are individually surrounded by a thin collagen layer called the *endomysium*. Individual muscle fibers aggregate to form muscle bundles, and each bundle is surrounded by a collagen layer called the *perimysium*. Muscle bundles combine to form whole muscles, which are surrounded by a third dense layer of collagen called the *epimysium* (Kranen et al., 2000; Velleman, 2020). The structural integrity of these layers of connective tissue is critical for the maintenance of muscle structure, and blood vessels and capillaries supplying muscle tissue with nutrients and oxygen run through the *perimysium* and *endomysium*, respectively (Kranen et al., 2000; Velleman, 2015).

Microscopically, a muscle fiber is composed of a succession of sarcomeres, the smallest structural and functional units in skeletal muscles. In normal *P. major* muscle, sarcomere lengths vary between 1.6 and 1.8 µm (Soglia et al., 2020). Sarcomeres are composed of thin and thick myofilaments, called actin and myosin myofilaments, respectively. These proteins represent the contractile fraction of muscle proteins (Mudalal et al., 2014). Muscle contraction is produced when actin filaments slide past myosin filaments, shortening the sarcomeres, which in turn leads to muscle fiber contraction (Squire, 2016). The cytoplasm of muscle fibers that fill the space between myofilaments is called the *sarcoplasm* and contains the sarcoplasmic fraction of muscle proteins. Anaerobic glycolysis, the main pathway that generates the necessary chemical energy to power muscle contraction, takes place in the sarcoplasm where glycogen reserves are stored and glycolytic enzymes are located (Xu and Becker, 1998; Meléndez-Morales et al., 2009).

Muscle fibers can be generally classified as type I and type II. Type II muscle fibers can be further classified into type IIA and type IIB (Talbot and Maves, 2016). The *P. major* muscle in commercial broiler chickens is entirely composed of the latter fiber type (Verdiglione and Cassandro, 2013). Compared with type I, type IIB muscle fibers have a larger diameter, faster contractile speeds, lower concentrations of myoglobin, and greater glycogen reserves (Huo et al., 2022). Metabolically, Type IIB muscle fibers have reduced numbers of mitochondria and large glycolytic reserves, which means anaerobic glycolysis is the main energy (*i.e.,* ATP) production pathway in this muscle (Velleman, 2020; Huo et al., 2022). This type of muscle fiber is adapted to short and intense bursts of activity, in contrast to type I fibers that are more adapted to less intense but longer periods of activity (Meléndez-Morales et al., 2009). Broilers are mostly kept in closed poultry houses (limiting the possibility of flight), which is likely the reason for the shift of their breast muscle fibers from type I, which is observed in the breast of their common ancestor the red jungle fowl (*Gallus gallus spadiceuse*), to type IIB fibers, which are observed in the breast muscles of modern broiler strains (Lokman et al., 2016). Selecting broilers for increased BMY is also a contributing factor to this switch in muscle fiber type as it has been previously shown that the mitochondrial content of the *P. major* muscle of broiler chickens was negatively correlated (−0.27, *p* = 0.037) with BMY (Reverter et al., 2017). Evidence suggests that in type IIB myofibers, centralized nuclei can be observed during growth in normal breast muscles (Rosser et al., 2002). According to these authors, this intermyofibrillar distribution of nuclei is an adaptation that allows muscle fibers to meet their requirements in protein synthesis during hypertrophic growth. This centralized position of nuclei coupled with the shift of mitochondria toward the periphery of myofibers during hypertrophic growth (Belichenko et al., 2004) indicate that type IIB myofibers are susceptible to becoming hypoxic at their core as the diffusion distance from blood capillaries increases due to increased myofiber diameter while demand for oxygen is simultaneously increasing due to the high metabolic rate of broiler chickens.

During embryogenesis, muscle development is achieved by the formation of embryonic myoblasts that fuse together to form myotubes, which later become mature muscle fibers (Yablonka-Reuveni, 1995; Greene et al., 2023). At hatch, the number of muscle fibers is fixed, and post-hatch muscle growth is achieved through the increase in muscle fibers size or myofiber hypertrophy (Remignon et al., 1995; Geiger et al., 2018). Muscle satellite cells are adult stem cells or precursor cells that are also formed during embryogenesis and located peripherally between the basal lamina and the sarcoplasmic membrane (or the sarcolemma) of muscle fibers (Daughtry et al., 2017). These cells provide the nuclei required for the hypertrophy of muscle fibers by fusing with them, and they also play a critical role in muscle repair (*i.e.,* regenerative myogenesis) after injury (Geiger et al., 2018; Velleman, 2023). Under normal conditions, satellite cells remain in a quiescent state in their niches. When stimulated, they activate and start proliferating to increase their number and to maintain their populations. They then differentiate to form new myofibers that replace degenerated muscle tissue (Velleman, 2015; 2020). In skeletal muscles, satellite cells need to be within 21 µm of blood capillaries to actively regenerate muscle fibers (Christov et al., 2007).

In summary, the *P*. *major* muscle in broiler chickens is composed of fast-twitch, hypertrophic, glycolytic muscle fibers using anaerobic glycolysis as the main energy production pathway. In case of injury or damage, satellite cells are activated to repair the damaged muscle tissue, which requires the presence of blood capillaries in the niches of these cells.

## 3 Consequences of muscle fiber hypertrophy in the *Pectoralis major* muscle

Genetic selection for increased growth rate and BMY is known to operate by increasing the diameter of muscle fibers. For instance, Geiger et al. (2018) compared two genetic lines of broiler chickens that were divergently selected over 58 generations for higher or lower body weight (BW), which is positively correlated with the growth of the *P. major* muscle (Alnahhas et al., 2014; Alnahhas et al., 2016). In the study of Geiger et al. (2018), the cross-sectional area of muscle (CSA) fibers in the *P. major* muscle from the line selected for increased BW was twice as large (*p* < 0.001) as that of the line selected for lower BW. In another study, Orlowski et al. (2021) compared the histomorphological traits of the *P. major* muscle at the 5th generation in two broiler lines divergently selected for greater (HBY4) or lower (LBY4) percent breast yield at 4 days of age. These authors showed that the diameter of muscle fibers of the *P. major* from the HBY4 line was significantly larger than that of the LBY4 line at day 56 (54.29 ± 1.13 vs. 45.39 ± 0.92 µm, *p* < 0.0001) while the number of muscle fibers in this muscle was not significantly different between the two lines. Finally, Koomkrong et al. (2015) compared a native Thai breed that did not undergo selection to a commercial strain of broiler chickens. These authors also reported a significantly greater diameter of muscle fiber in the *P. major* muscle of the commercial strain compared with that of the native breed (52.31 ± 1.83 vs. 33.31 ± 2.20 µm, *p* = 0.003). These findings provide further evidence that muscle fiber hypertrophy in broiler breast muscles is the primary driver of post-hatch growth, and it is mainly induced by selective breeding for increased BW and BMY.

### 3.1 Muscle fiber hypertrophy is associated with decreased muscle capillary density

One consequence of myofiber hypertrophy is the decrease in connective tissue spaces, especially in the *perimysium* and *endomysium* (Velleman et al., 2003; Velleman, 2019). As mentioned earlier, muscle blood capillaries run through these layers of connective tissue. Thus, a reduction in their spaces leads to reduced muscle capillary density. For instance, Joiner et al. (2014) evaluated the histomorphology of the *P. major* muscle from standard (2.5 kg) and heavy (3.5 kg) broilers and reported a significant increase in the diameter of muscle fibers (+18%) that was associated with a significant decrease in the average number of capillaries (−22.9%) and blood vessels (−43.9%) in the heavy broilers compared to the standard broilers. More recently, Pampouille et al. (2019) compared the histological characteristics of a normal *P. major* muscle from a slow-growing genetic line (similar to the French Label Rouge) to normal *P. major* muscle from a fast-growing commercial line of broiler chickens and showed that increased growth rate and BMY were associated with a 121% (*p* < 0.001) increase in muscle fiber size coupled with a 34% (*p* < 0.001) decrease in the number of blood capillaries. Yalcin et al. (2019) also compared slow- and fast-growing commercial broilers at market age and reported a 48% increase (*p* < 0.05) in myofiber area that was associated with a 14.9% decrease in the capillary density in the *P. major* muscle of the fast-compared to the slow-growing strain. The reduced capillary density of the *P. major* muscle in fast-growing and high-yielding broiler strains is the primary factor predisposing this muscle to the development of BMM as it will be discussed in the following sections.

### 3.2 Muscle fiber hypertrophy is associated with decreased muscle energy reserves

Another consequence of genetic selection for increased BW and BMY is decreased energy (glycogen) reserves in the *P. major* muscle. In an earlier study, Berri et al. (2001) compared an experimental and a commercial line to their respective control lines in terms of glycolytic potential at 6 weeks of age. These authors showed a significant (*p* < 0.05) decrease in the glycolytic potential from 128 to 87 μM/g in the *P. major* muscle from the selected commercial line compared with its non-selected control line. In a later study, Berri et al. (2007) investigated the histological and metabolic characteristics of the *P. major* muscle of male birds from a commercial grand-parental line of broiler chickens at 6 weeks of age. In this study, muscle fibers were grouped into five categories based on their CSA ranging from 1,260 to 2,443 μm^2^ before comparing their metabolic characteristics. Findings from this work demonstrated that muscle energy reserves, as measured by the glycolytic potential, decreased significantly from 111.6 to 102.4 μM/g with increased muscle fiber CSA.

**FIGURE 1:**
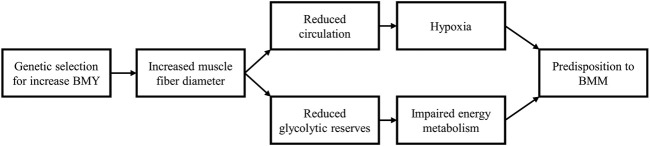
Consequences of muscle fiber hypertrophy. Genetic selection for increased breast meat yield (BMY) operates by increasing muscle fiber diameter. Myofiber hypertrophy leads to a decrease in the interstitial spaces (*perimysium* and *endomysium*) in which blood vessels and capillaries are found, resulting in a reduced density of the vascular network. Myofiber hypertrophy is also associated with decreased energy or glycogen content in muscle tissue. The reduced capillary density and decreased glycogen reserves predispose the *Pectoralis major* muscle to the development of breast muscle myopathies.

In summary (Figure 1), increased muscle fiber diameter reduces the capillary density and glycogen storage in the *P*. *major* muscle compromising its functioning and predisposing it to the occurrence of BMM.

## 4 Breast muscle myopathies (BMM)

Selective breeding for increased BW and BMY has resulted in structural and metabolic changes in the *P*. *major* muscle. These changes have led to the emergence of a new category of non-infectious myopathies collectively called breast muscle myopathies (Petracci and Cavani, 2012; Petracci et al., 2015; Kuttappan et al., 2016; Barbut, 2019). The three most commonly known and studied myopathies are WS, WB, and SM. These myopathies were first reported in 2009, 2014, and 2016 by Bauermeister et al. (2009), Sihvo et al. (2014) and by Sirri et al. (2016), respectively. In this section, we will briefly describe the pathological changes associated with these myopathies in the *P. major* muscle before we described the role of hypoxia in the development of these changes in the following sections.

### 4.1 Macroscopic description of myopathic muscles

**FIGURE 2:**
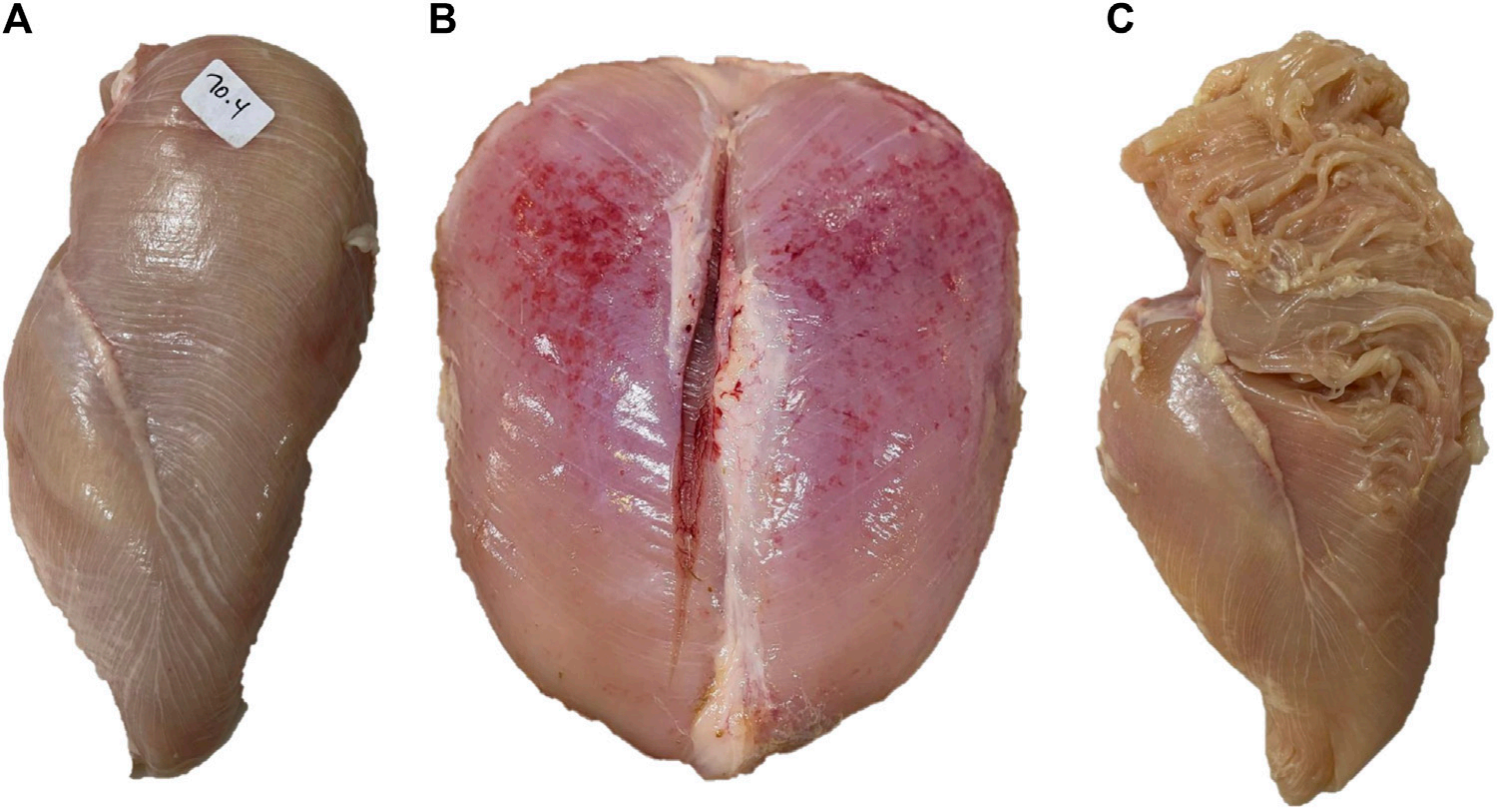
Examples of breast muscles exhibiting severe white striping **(A)**, wooden breast **(B)** and spaghetti meat **(C)**. Severe white striping is characterized by the occurrence of white striations with a diameter greater than 1 mm, running in parallel to the direction of muscle fibers, covering the entire ventral surface of the muscle. Severe wooden breast is associated with increased muscle hardness from the cranial to the caudal end of the *Pectoralis major* muscle, coupled with petechia. Severe spaghetti meat is associated with disintegration and separation of muscle bundles appearing like spaghetti.

BMM have very distinctive visual and textural characteristics. White striping (WS, Figure 2A) can be described as the appearance of white striations running parallel to the direction of muscle fibers on the ventral (skin) side of the *P. major* muscle (Kuttappan et al., 2012). The thickness of these striations and the muscle surface they cover depend on the severity of this myopathy. In mild to moderate forms, the thickness of the striations is usually less than 1 mm and cover the cranial part of muscle surface, while in severe cases, their thickness is greater than 1 mm and cover the entire ventral surface of the muscle (Kuttappan et al., 2012; Khalil et al., 2021).

Wooden breast (WB, Figure 2B), as its name implies, is characterized by increased muscle hardness. In the mild forms of WB, this hardness is rather focal and limited to the cranial part of the *P. major* muscle, while in severe cases, it diffuses toward the caudal part of the muscle leading to extensive hardness of the entire muscle accompanied by bulges and petechiae covered with a thin viscous layer of clear or turbid material (Sihvo et al., 2014).

Spaghetti meat (SM, Figure 2C) is described as the loss of cohesiveness in the tissue of the *P. major* muscle (Sirri et al., 2016). In its moderate forms, it is characterized by a moderate loss of cohesiveness on the cranioventral part of the muscle leading to a loose muscle tissue that can fold relatively easily when pinched. In its severe forms, extended superficial lacerations of muscle tissue can be observed on the cranioventral surface of the muscle (Sirri et al., 2016). In addition, muscle bundles are separated and appear as thin mushy fibers that resemble spaghetti, hence the name (Baldi et al., 2021).

### 4.2 Microscopic description of myopathic muscles

BMM are characterized by multiphasic degenerative processes that induce regenerative and anti-inflammatory responses from muscle tissue. In the case of WS, Kuttappan et al. (2013) reported muscle fiber degeneration, lysis, and loss of cross-striation in the *P. major* muscle of 45-day-old commercial broiler chickens. They also reported multifocal edema and infiltration of lymphocytes and macrophages in the interstitial spaces of these muscles. Signs of muscle tissue regeneration such as variability in muscle fiber size, nuclear rowing and the presence of multinucleated cells were also reported in this study. One of the distinctive features of WS is increased lipid deposition (lipidosis) within the connective tissue, leading to the appearance of the characteristic phenotype of white striations on the surface of the muscle (Alnahhas et al., 2016; Baldi et al., 2018).

WB-affected *P. major* muscles exhibit similar histopathological changes to those observed in WS. For instance, Sihvo et al. (2014) reported multifocal degeneration of muscle fibers, loss of cross-striation, and infiltration of inflammatory cells including macrophages and heterophils within and around the degenerated muscle fibers in the *P. major* muscle from 5 to 6-week-old commercial broiler chickens. These authors also reported the presence of muscle fibers of variable diameters and centralized nuclei, which are signs of regeneration of damaged muscle tissue. One of the most important characteristics of WB is the diffuse thickening of the interstitial spaces with a variable amount of collagen-rich connective tissue or fibrosis (Sihvo et al., 2014). This replacement of muscle tissue by fibrous connective tissue leads to the increased hardness of the *P. major* muscle that can be detected by palpation (Velleman et al., 2017).

SM also presents histopathological changes similar to those associated with WS and WB. According to Baldi et al. (2018), SM-affected *P. major* muscles are very soft and stringy, particularly in the cranioventral part. These authors reported extensive myofiber degeneration accompanied by inflammatory cell infiltration, lipidosis in damaged muscle tissue, and regeneration of fibers with variable diameters. The distinctive characteristics of SM are the compromised (*i.e.,* very thin) connective tissue of the *perimysium* and *endomysium,* and the numerous longitudinally split myofibers that give this myopathy its specific phenotype (Baldi et al., 2018).

The histopathological changes associated with BMM are more pronounced in the cranial and middle part of the *P. major* muscle (Che et al., 2022), and are mostly focused in the superficial layer (0.5–1.2 cm from the ventral surface) of this muscle in WS (Baldi et al., 2018) and in both the superficial and deep (from 1.5 to 2.5 cm from the ventral surface) layers of the muscle in WB (Soglia et al., 2017).

### 4.3 Protein, lipid, and collagen content of myopathic muscles

The histopathological changes associated with BMM induce changes in the chemical composition of the *P. major* muscle. Gratta et al. (2019) investigated the effect of WS and WB on the proximate composition of the *P. major* muscle from 49-day-old commercial broiler chickens. In this study, protein content was similar in WS-affected fillets and normal fillets (23.2% vs. 23.9%, *p* > 0.05) while the protein content in WB was significantly lower than in normal fillets (21.4% vs. 23.9%, *p* < 0.05). Baldi et al. (2019) analyzed the proximate composition of the superficial and deep layers of normal and myopathic *P. major* muscles from 46-day-old commercial broiler chickens. Compared with normal fillets, WB- and SM-affected fillets had significantly (*p* < 0.001) lower protein content (20.5, 21.0, and 22.9% for WB, SM, and normal, respectively) in the superficial layer, while in the deep layer, only SM-affected fillets had a lower protein content than unaffected muscles (21.6% vs. 23.0%, *p* < 0.001). In both layers, the protein content of WS-affected fillets was not significantly different from normal fillets (22.0% vs. 22.9% and 22.5% vs. 23.0%, respectively, for the two layers). Fillets affected with any of the three myopathies had significantly (*p* < 0.05) greater lipid content in the superficial layer than normal fillets (1.51, 2.05, 2.12, and 1.84% for normal, WS, WB, and SM, respectively). In this study, the same trend (*p* < 0.05) was also found in the deep layer of the *P. major* muscle (1.59, 1.84, 1.80, and 1.83% of lipid for normal, WS, WB, and SM, respectively). Taken together, these findings indicate a more extensive degenerative process that can reach the deep layers of the *P. major* muscle in the case of WB and SM, but it tends to be more focused in the superficial layer in the case of WS in line with the above-reported histopathological changes.

In skeletal muscles, collagen is one of the main building blocks of the connective tissue that maintains the structural integrity of these muscles (Sanden et al., 2021). As previously mentioned, BMM are associated with fibrosis, where degenerated or necrotic muscle tissue is replaced by fibrous collagen-rich tissue, resulting in the development of the BMM phenotypes such as muscle hardness in WB-affected fillets. There are two types of collagen fibrils in skeletal muscles: type I and type III. This latter type is predominant in muscles during early growth stages and after injuries (Velleman, 2020). In myopathic muscles, the distribution of type III collagen was found to vary between WS- and WB-affected muscles (Mazzoni et al., 2020). Using immunohistochemistry on breast muscle samples from 45-days-old fast-growing broilers, these authors showed that the connective tissue of WS-affected muscle was strongly reactive for collagen type III while a weak immunoreactivity to this collagen fibril type was found in the perimysium and endomysium of WB-affected muscles. Multiple studies have investigated changes in collagen content in myopathic muscles. In their work, Soglia et al. (2016a) compared fillets from 52-day-old commercial broilers exhibiting WB, WS, or both (WB + WS) with normal fillets and reported a significant (*p* < 0.001) increase in collagen content in (WB + WS)–affected fillets compared with normal fillets (1.09 < 1.18 < 1.26%, respectively for normal, WB, and WB + WS). Geronimo et al. (2022) compared the ratio of collagen-to-protein between normal and WB-affected *P. major* muscles from 47-day-old commercial broilers and saw a significant increase in this ratio in the WB-affected muscles compared with normal muscle (7.15 vs. 5.18, *p* < 0.05). Findings from these two studies clearly showed that with increased severity of WB, muscle tissue is replaced by fibrous tissue, which explains the increased hardness of WB-affected muscles. This statement is also supported by the work of Ferreira et al. (2020) who used fluorescent Western blotting to analyze the relative expression of collagen protein in the *P. major* muscle sampled from a high-yielding strain of broiler chickens at the age of 43 days, scoring them as normal, mild, and severe with respect to WB. These authors found that the relative expression of collagen protein increased (*p* = 0.001) with the severity of WB (3-fold increase from normal to severe) at 43 days of age. Evidence suggests that different changes in the structural organization of muscle collagen could lead to the development of phenotypically distinctive myopathies. For instance, Sanden et al. (2021) combined differential staining and Fourier Transform Infrared (FTIR) microspectroscopy to study changes in the collagen structure of the *P. major* muscle from 32-day-old commercial broilers exhibiting WB and SM. In cross-sections of the muscle stained with picrosirius red, the *perimysium* of myopathic muscles was heterogeneously stained, and it exhibited gaps and short threads oriented in different directions. This is in contrast to the *perimysium* of normal muscles, which appeared as compact and continuous strings with evenly sized, parallelly aligned, and tightly packed bundles of collagen. Furthermore, the matrix was denser and more intensely stained in WB-affected muscles than in SM-affected muscles. According to Sanden et al. (2021), these changes suggest alterations in the structure and organization of collagen fiber in myopathic muscles. An interesting finding from this study was that collagen from SM-affected muscles was rich in loosely bound *a*-helices, while WB-affected fillets had more triple helical and *ß*-sheet structures. This could explain the difference between their distinctive phenotypes of structural disintegration or extreme hardness, respectively.

In summary, the development of BMM is associated with the replacement of muscle tissue by increased amounts of lipids and disorganized connective tissue, which contributes to the development of their characteristic phenotypes.

### 4.4 Biochemical characteristics of myopathic muscles

The hypertrophic growth of breast muscles of fast-growing and high-yielding strains of broiler chickens is associated with a decrease in glycogen reserves (Berri et al., 2007; Abasht et al., 2016), resulting in a higher ultimate pH of breast meat in severe cases of BMM (Mudalal et al., 2015). Baldi et al. (2020) conducted a study that aimed to elucidate the mechanisms underlying the higher ultimate pH in WB-affected *P. major* muscles sampled from 48-day-old commercial broilers. As expected, muscle glycogen levels were lower (*p* < 0.001) in WB-affected muscles compared with normal muscles at 15 min *post-mortem*. The ultimate pH (pHu) is almost completely determined by the glycolytic potential (GP) of the *P. major* muscle at death as measured by the GP at 15 min *post-mortem*, and these two parameters (GP and pHu) were found to be almost perfectly negatively correlated at the genetic level (Le Bihan-Duval et al., 2008). Consequently, in the study of Baldi et al. (2020), the lower GP at 15 min *post-mortem* led to a significantly higher (*p* < 0.001) pHu in WB-affected muscles at 24 h *post-mortem*, which could be attributed to an earlier cessation of *post-mortem* acidification caused by the lower initial (at death) glycogen content in WB-affected muscles. Additionally, in WB-affected muscles, Zhao et al. (2020) reported a 2.8-fold (*p* < 0.02) and 3.5-fold (*p* < 0.004) increase in the mRNA and protein expression of monocarboxylate transporter 4 (*MTC4*) in WB-affected muscles compared with normal muscles. This translates to an increase in lactate exportation from the cell (Zhao et al., 2020), which could also contribute to the higher pHu in WB-affected muscles. In their study, Baldi et al. (2020) suggested multiple hypotheses to explain the changes in *post-mortem* energy metabolism in myopathic muscles. One of the hypotheses suggested an impaired mitochondrial function based on the significantly (*p* < 0.001) lower ATP levels found in WB-affected muscles at 15 min *post-mortem*. In a recent study, Li et al. (2022) examined the effect of WB severity on the activity of citrate synthase, a major enzyme in the tricarboxylic acid cycle (TCA) and a marker of mitochondrial activity. In support of the hypothesis of Baldi et al. (2020), Li et al. (2022) showed a significant (*p* < 0.05) decrease in the activity of this enzyme from 2.7 µmol/(mLmin) in normal *P. major* muscle to 1.8 µmol/(mLmin) in severely affected muscles. This finding, coupled with the observed deterioration of mitochondrial ultrastructure (Sihvo et al., 2018; Hosotani et al., 2020), could also contribute to the higher pHu in WB-affected muscles. This mitochondrial damage may potentially reduce *in vivo* ATP synthesis, resulting in rapid ATP depletion and in the cessation of *post-mortem* metabolism shortly after death.

**FIGURE 3:**
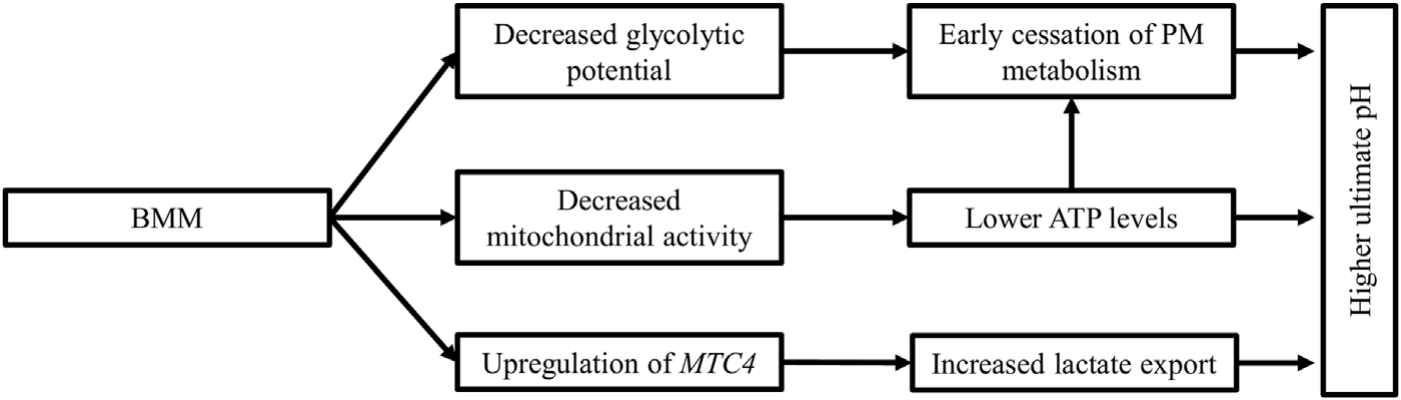
Biochemical changes in myopathic muscles. Breast muscle myopathies are associated with a higher ultimate pH than unaffected muscles. The increase in ultimate pH can be explained by the lower glycolytic reserves in these muscles at death, coupled with decreased mitochondrial activity, leading to lower ATP levels and early cessation of *post-mortem* acidification. The lower lactate level in myopathic muscles is the result of the upregulation of the monocarboxylate transporter 4 (*MTC4*), which increases lactate export from cells.

To summarize (Figure 3), myopathic muscles are characterized by a deficient energy reserve and perturbated energy production leading to early cession of *post-mortem* acidification and subsequently a higher ultimate pH in the *P. major* muscle.

## 5 Hypoxia induces the development of breast muscle myopathies

The immediate consequences of the reduced density of the capillary network in the *P. major* muscle (see section 3.1) and the subsequent reduction in circulation are hypoxia, lower nutrient supply, and accumulation of metabolic waste in muscle tissue which are all factors that predispose muscles for the development of BMM.

Hypoxia is now largely accepted as a trigger of the development of BMM (Zambonelli et al., 2016; Boerboom et al., 2018; Greene et al., 2019; Emami et al., 2021). In their study, Greene et al. (2019) used diffused reflectance spectroscopy to measure oxygen saturation in the *P. major* muscle of WB-affected and unaffected commercial broilers. These authors found a significant decrease in oxygen saturation in the thickest part of the severely WB-affected muscles, which is indicative of a hypoxic condition. Emami et al. (2021) placed random-bred modern broilers in hypobaric chambers to simulate high altitude (*i.e.,* lower oxygen) conditions from 2 to 6 weeks of age. In this study, the total incidence of WB in the birds kept under high altitude conditions was 3.3-fold higher than that of birds kept under low altitude conditions (90.41% vs. 27.69%) at similar BW. These two studies provided experimental evidence confirming the critical role of hypoxia in the development of BMM.

Under hypoxic conditions, the HIF-1 pathway, a major regulator of oxygen homeostasis, is upregulated (Greene et al., 2019). In this section, the consequences of the upregulation of this pathway on the development of BMM will be examined after a brief overview of the HIF-1 pathway.

### 5.1 Overview of the hypoxia-inducible factor 1 (HIF-1) pathway

**FIGURE 4:**
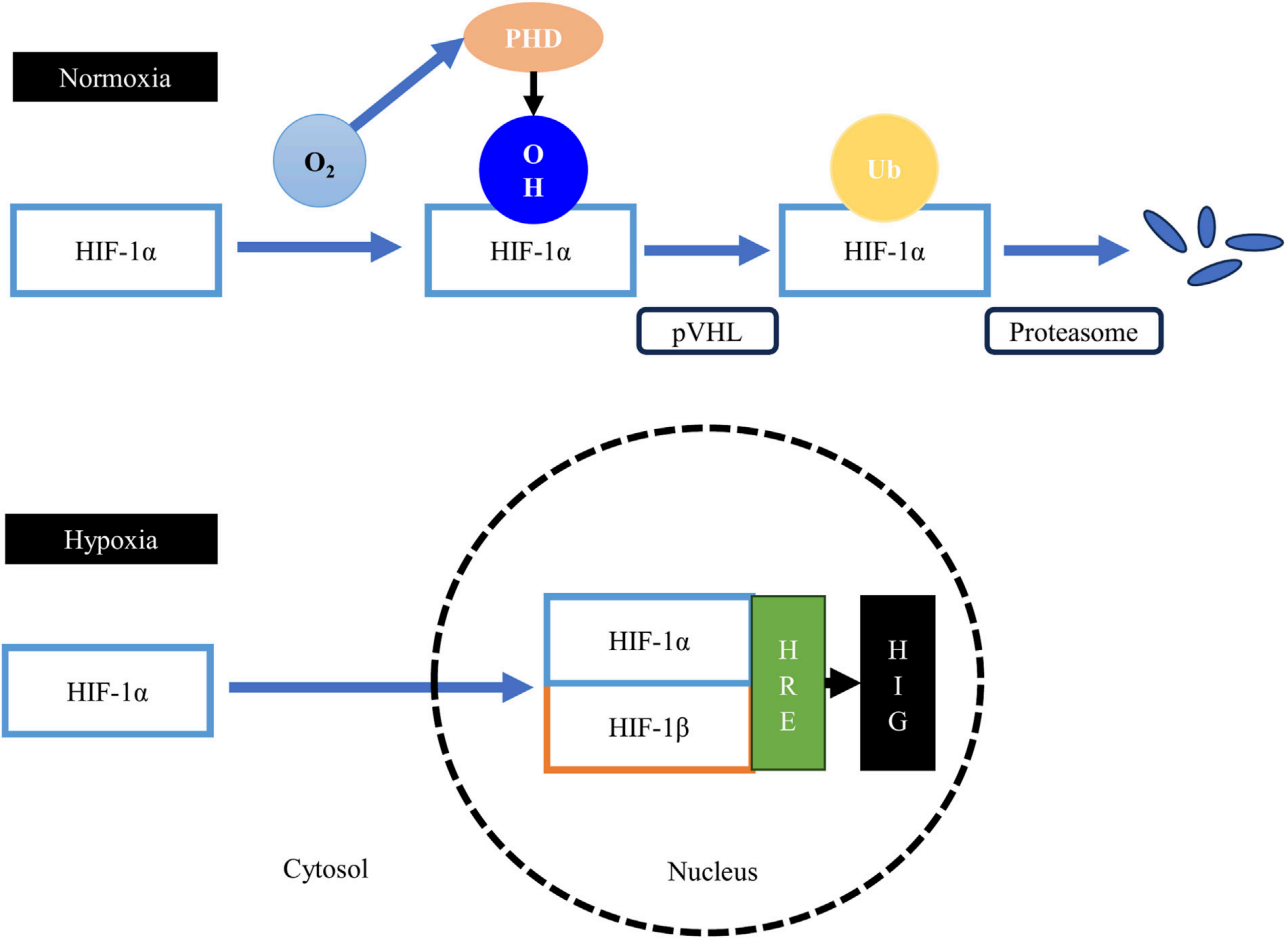
The hypoxia-inducible factor 1 (HIF-1) pathway. Under normoxic conditions, the proline residues on the HIF-1α subunit are hydroxylated by the oxygen-dependent prolyl-4-hydroxylases (PHD), leading to the binding of the von Hippel–Lindau tumor suppressor protein (pVHL), the substrate recognition component of an E3 ubiquitin ligase, to the HIF-1α protein. The pVHL protein tags the sarcoplasmic HIF-1α protein for ubiquitination and subsequent rapid proteasomal degradation. Therefore, formation of the HIF-1 heterodimer is obstructed, suppressing the transcription of hypoxia-inducible genes (HIG). Under hypoxic conditions, the activity of the prolyl-4-hydroxylases is suppressed, which allows the stabilization of the HIF-1α subunit and its subsequent translocation to the nucleus to form the HIF-1 heterodimer with the HIF-1β subunit. The heterodimer then binds to the hypoxia-responsive elements (HRE), upregulating the expression of HIG.

HIF-1 (Figure 4) is a heterodimeric transcription factor consisting of two subunits: the *a* (HIF-1α) and *ß* (HIF-1β) subunits (Ziello et al., 2007). In contrast to HIF-1β, which is constitutively expressed, HIF-1α is oxygen sensitive or oxygen regulated (Masoud and Li, 2015).

Under normoxic conditions, HIF-1α is continuously synthesized, but it has a very short lifespan as cells continuously degrade HIF-1α protein (Weidemann and Johnson, 2008). The *a* subunit of HIF-1 has a degradation domain called the oxygen-dependent degradation domain (Huang et al., 1998). This domain has two proline residues (Pro^402^ and Pro^564^) that are hydroxylated under normoxic conditions by a group of oxygen-dependent enzymes called prolyl-4-hydroxylases or PHD (Kant et al., 2013). The hydroxylation of these two proline residues lead to the binding of the von Hippel–Lindau tumor suppressor protein (pVHL), the substrate recognition component of an E3 ubiquitin ligase, to the HIF-1α protein (Haase, 2009). The pVHL protein tags the cytoplasmic HIF-1α protein for ubiquitination and subsequently for rapid proteasomal degradation (Haase, 2009). Thus, when oxygen is available in the cells, the HIF-1 transcription factor cannot be formed, and consequently, hypoxia-inducible genes are not transcribed.

Under hypoxic conditions, the hydroxylation of the proline residues on the cytoplasmic HIF-1α protein cannot take place, because PHD enzymes are oxygen-dependent and are suppressed during the absence of oxygen (Jaakkola et al., 2001). The persisting HIF-1α protein in the cytoplasm can then be translocated to the nucleus to form the HIF-1 heterodimer with the HIF-1β subunit. This transactivating complex can then bind to hypoxia-responsive elements (HRE) in hypoxia-inducible genes (HIG), leading to their enhanced transcription (Weidemann and Johnson, 2008).

As a major transcription factor, HIF-1 not only regulates oxygen homeostasis, but it is also involved in energy metabolism, including glycolysis, oxidative phosphorylation and fatty acid metabolism (Weidemann and Johnson, 2008; Kierans and Taylor, 2021), angiogenesis, apoptosis, and several other pathways of importance to BMM (Malila et al., 2019; Marchesi et al., 2019). Finally, HIF-1 is involved in the regulation of mitochondrial mass, size, distribution, and morphology (Thomas and Ashcroft, 2019) and also in the regulation of mitochondrial dynamics (Hosotani et al., 2020).

### 5.2 *HIF-1α* is upregulated in myopathic muscles

Hypoxia plays a key role in the development of BMM. In this context, multiple studies have reported an upregulation of the HIF-1 pathway in muscles exhibiting one or more myopathies. In their study, Marchesi et al. (2019) analyzed the whole transcriptome of WS-affected *P. major* muscles from 42-day-old commercial broiler chickens using the RNA-Seq technology. Among the differentially expressed genes detected in this study, *HIF-1α* was upregulated (>1.2-fold) in WS-affected muscles when compared with normal muscles. Using droplet digital PCR, the mRNA expression of *HIF-1α* was investigated in normal and myopathic muscles exhibiting WS or WS + WB from 6-week-old broilers with medium- or heavy-weight carcasses (Malila et al., 2019). This study also demonstrated an approximate 4-fold increase in the expression of *HIF-1α* in the myopathic muscles compared with normal muscles. In a different study, the expression of *HIF-1α* at the mRNA and protein levels was measured in WB-affected muscles sampled from 56-day-old broilers using RT-qPCR and Western blotting, respectively (Greene et al., 2019). These authors confirmed the increased expression of *HIF-1α* at the mRNA level in the myopathic muscles compared with unaffected muscles, and further showed a significant increase in its expression at the protein level.

Taken together, these findings provide further evidence for the critical role of hypoxia in the occurrence of BMM and highlight the key role of the HIF-1 pathway in the development of these myopathies.

### 5.3 Hypoxia alters energy metabolism in myopathic muscles

The HIF-1 pathway plays a key role in the adaptation of cells to hypoxia by promoting a shift in their energy metabolism from mitochondrial respiration toward anaerobic glycolysis (Kierans and Taylor, 2021). As discussed previously, the *P. major* muscle of broiler chickens is entirely composed of type IIB myofibers (Verdiglione and Cassandro, 2013), in which anaerobic glycolysis is the predominant energy production pathway. This is important to highlight because, in skeletal muscles, the expression of *HIF-1α* varies according to muscle fiber type. At the mRNA level, the expression of *HIF-1α* in mouse glycolytic muscles (composed of type IIB myofibers) was 3-fold higher than that in oxidative muscles (composed of type I myofibers), while at the protein level, the expression of HIF-1α was 8- to 10-fold higher in the glycolytic than in the oxidative muscles (Pisani and Dechesne, 2005). Thus, glycolytic muscles are expected to have a degree of hypoxia under physiological conditions that favor anaerobic glycolysis over aerobic phosphorylation. However, in the case of BMM, a pronounced lack of oxygenation is observed when compared with unaffected breast muscles which alters energy metabolism.

#### 5.3.1 Hypoxia further promotes anaerobic glycolysis in myopathic muscles

Myopathic muscles have lower glycogen content than normal muscles (Abasht et al., 2016; Baldi et al., 2020). One hypothesis to explain this finding is chronic hypoxia. With age (*i.e.,* increased muscle growth and development), the diameter of muscle fibers increases while the number of capillaries decreases, which leads to a lower myofiber surface area being exposed to capillaries, subsequently reducing oxygen supply (Sihvo et al., 2018) and increasing the diffusion distance of critical micronutrients from the capillaries to the myofibers (Joiner et al., 2014). Under such conditions, hypoxia can further stimulate anaerobic glycolysis as birds grow to meet muscle energy requirements, which consequently depletes the glycolytic reserves as birds reach market age and dysregulates *post-mortem* energy metabolism (see section 4.4). The hypothesis of chronic hypoxia is supported by findings from the literature showing that the expression of *HIF-1α* increased (*p* < 0.05) progressively with age from 21 to 42 days in the *P. major* muscle of fast-growing broiler chickens (Young and Rasmussen, 2020; Malila et al., 2022).

In myopathic muscles, stimulated anaerobic glycolysis is associated with increased conversion of pyruvate to lactate which is also mediated by HIF-1α. For instance, the increase in the mRNA expression of *HIF-1α* (2.0-fold) has been shown to be associated with an increase (1.5-fold) in the mRNA expression of the gene encoding pyruvate dehydrogenase kinase 1 (*PDK1*) in WB-affected muscles from 7-week-old commercial broilers (Thanatsang et al., 2020). Under hypoxic conditions, *HIF-1α* directly activates the gene encoding the PDK1 enzyme, which in turn inactivates pyruvate dehydrogenase through phosphorylation. This results in reduced conversion of pyruvate to acetyl-CoA to limit oxygen consumption while continuing to fulfill cellular energetic requirements (Kim et al., 2006). Concomitantly, *HIF-1α* upregulates the expression of lactate dehydrogenase (*LDH*), which mediates the conversion of pyruvate to lactate (Cui et al., 2017). Accordingly, the abundance of LDH in myopathic muscles exhibiting WB/WS has been confirmed at the protein level using SDS-PAGE (Zambonelli et al., 2016), and pyruvate concentration was found to be significantly lower in WB-affected muscles than in unaffected muscles (Abasht et al., 2016). Paradoxically, lactate levels in WB-affected muscles have been shown to decrease rather than increase (Abasht et al., 2016; Baldi et al., 2020). As mentioned before, *HIF-1α* upregulates the monocarboxylate transporter 4 (*MCT4*) responsible for lactate export from cells under hypoxic conditions (Ullah et al., 2006). In WB-affected muscles, the expression of *MTC4* is significantly upregulated at both the mRNA and protein levels (Zhao et al., 2020), which can partly explain the lower lactate levels and the subsequently higher pHu in myopathic muscles (Baldi et al., 2020).

#### 5.3.2 Hypoxia inhibits the TCA cycle in myopathic muscles

Under normoxic conditions, pyruvate is transported from the sarcoplasm to the mitochondria where it is used to produce acetyl-CoA (Martínez-Reyes and Chandel, 2020). Acetyl-CoA is then used in the TCA cycle to generate electrons that are transported in the form of NADH and FADH_2_ to the electron transport chain (ETC). These electrons establish a proton gradient across the inner mitochondrial membrane, which is then used by ATP synthase to produce ATP (Taylor and Scholz, 2022). As discussed in the previous section, by increasing PDK1 levels, HIF-1α inhibits the TCA cycle by reducing the conversion of pyruvate to acetyl-CoA. Furthermore, myopathic muscles affected by WS or WB have been shown to contain elevated levels of fumarate, malate, and citrate (Abasht et al., 2016; Boerboom et al., 2018). These TCA intermediates play a regulatory role in the HIF-1 pathway and have been found to inhibit the prolyl-4-hydroxylases (Koivunen et al., 2007). As mentioned earlier, the inhibition of these enzymes leads to the stabilization of the *a*-subunit of HIF-1 in the sarcoplasm and subsequently its translocation into the nucleus to form the HIF-1 complex with the *ß* subunit. In turn, this enhances the transcription of hypoxia-responsive genes and pathways, including *PDK1*, which further reduces the conversion of pyruvate to acetyl-CoA and inhibits the TCA cycle to reduce oxygen consumption and enhance cell survivability. This could partly explain the lower ATP levels reported in WB-affected muscles at 15 min *post-mortem* (Baldi et al., 2020).

**FIGURE 5:**
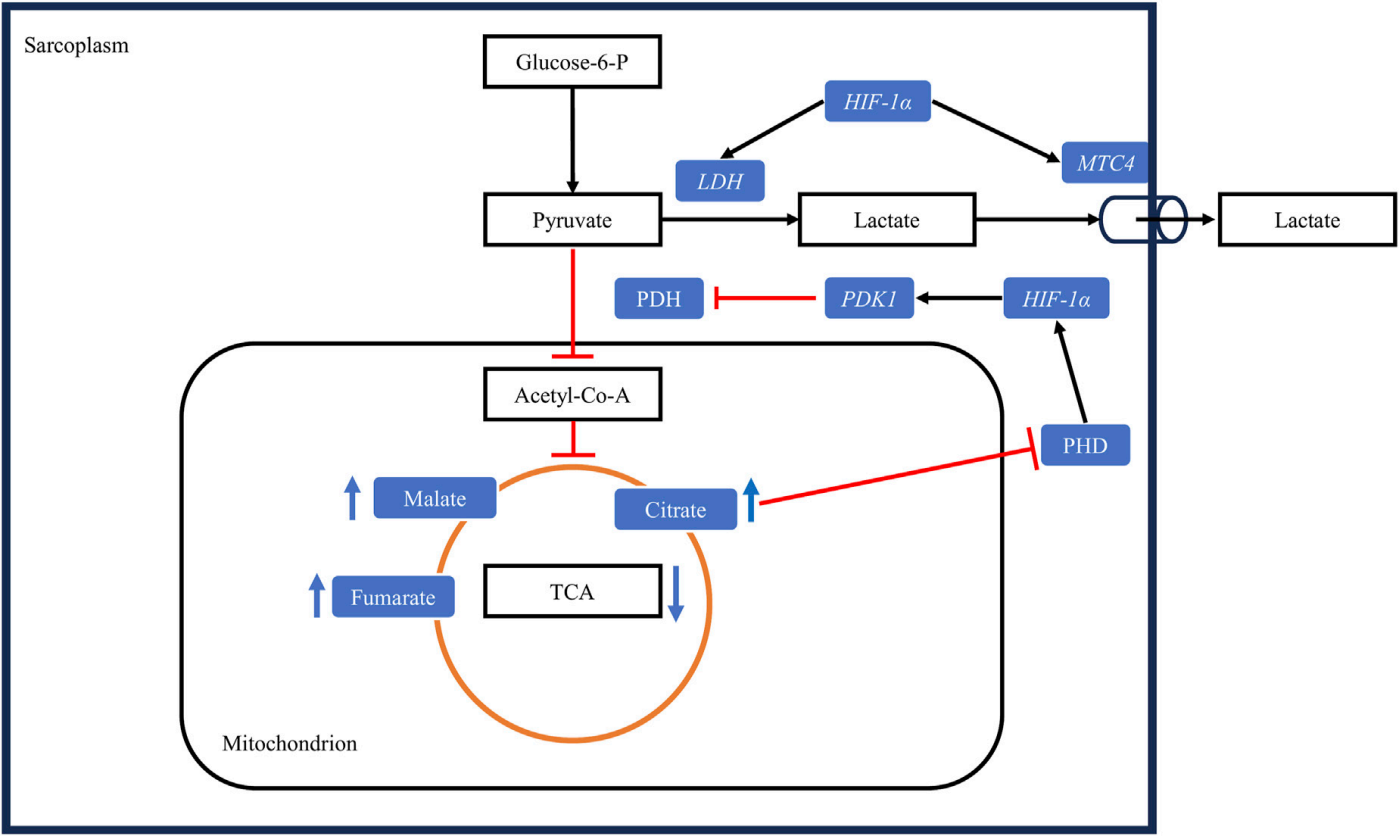
Hypoxia alters muscle energy metabolism through the upregulation of the HIF-1 pathway. Under hypoxic conditions, *HIF-1α* upregulates pyruvate dehydrogenase kinase 1 (*PDK1*), leading to the inactivation of the enzyme pyruvate dehydrogenase (PDH) through phosphorylation. Consequently, the conversion of pyruvate to acetyl-CoA is decreased. Concurrently, *HIF-1α* upregulates lactate dehydrogenase (*LDH*), increasing the conversion of pyruvate to lactate, and upregulates the monocarboxylate transporter 4 (*MTC4*), promoting lactate export from cells. The decrease in the conversion of pyruvate to acetyl-CoA slows the tricarboxylic acid cycle (TCA), leading to the accumulation of its intermediates. These intermediates downregulate the activity of prolyl-4-hydroxylases (PHD), further upregulating the HIF-1 pathway by allowing the stabilization of HIF-1α protein in the sarcoplasm. Black arrows: upregulation, blunt red arrows: downregulation.

In summary (Figure 5), activation of the HIF-1 pathway in myopathic muscles can lead to reduced glycogen content in the *P. major* muscle at market age, inhibition of the TCA cycle, increased conversion of pyruvate to lactate and increased export of lactate from cells, resulting in higher pHu in these muscles.

### 5.4 Hypoxia alters lipid metabolism in myopathic muscles

Myopathic breast muscles are also characterized by dysregulated lipid metabolism (Liu et al., 2022; Boerboom et al., 2023), which causes increased lipid accumulation in muscle tissue (Alnahhas et al., 2016; Baldi et al., 2019). Hypoxia can contribute to lipid accumulation in myopathic muscles through increased fatty acid synthesis, adipogenesis, and lipid accumulation. With regard to fatty acid synthesis, the upregulation of *HIF-1α* under hypoxic conditions has been shown to downregulate the expression of myogenic regulatory factors, including *Pax7* (paired box-7) and *Myf5* (myogenic factor-5), and to upregulate the expression of lipogenic factors, including *ACCα* (acetyl-CoA carboxylase) and *FAS* (fatty acid synthase), at the mRNA and protein level in WB-affected muscles (*in vivo*) and in satellite cells (*in vitro*) from broiler breast muscles (Emami et al., 2021). ACCα is a major rate-limiting enzyme in the biosynthesis of long-chain fatty acids, as it catalyzes the carboxylation of acetyl-CoA to form malonyl-CoA (Tian et al., 2010), which is then used by FAS to synthesize long-chain fatty acids. In normal breast muscles, fatty acid synthesis is almost nonexistent, because these muscles lack the enzymes required for this process, particularly FAS (Cui et al., 2012). Thus, elevated expression levels of *ACCα* and *FAS* in satellite cells under hypoxic conditions suggest that these cells switched from their myogenic program to a lipogenic program, leading to increased fatty acid synthesis and accumulation in hypoxic muscles (Emami et al., 2021). This statement is supported by findings from metabolomic studies showing significantly higher levels of long-chain fatty acids in WB-affected (Abasht et al., 2016) and WS-affected (Boerboom et al., 2018) muscles.

Lipid accumulation involves the peroxisome proliferator-activated receptor gamma (PPARγ), which is a ligand-dependent transcription factor, a master regulator of adipogenesis, and an active modulator of lipid metabolism (Ma et al., 2018). The expression of the *PPARγ* gene was upregulated in WB-affected muscles from 7-week-old commercial broiler chickens (Lake et al., 2019). These authors argued that the increased expression of this gene could induce a pathological deposition of lipids in WB-affected muscles, promoting lipid accumulation in myopathic muscles. In a more recent study, the expression of *PPARγ* was investigated *in vitro* using satellite cells from the breast muscles of a random-bred line and from a modern line of broiler chickens (Velleman et al., 2022). These authors showed that the expression level of *PPARγ* was significantly higher in the modern line than in the random-bred line. More importantly, they showed that knocking down *PPARγ* was associated with reduced lipid accumulation in satellite cells from both lines during the proliferation phase of these cells, which supported the role of this gene in lipid accumulation in myopathic muscles. Hypoxia was shown to upregulate *PPARγ* in a human hepatocellular carcinoma cell line (Zhao et al., 2014) and in cardiomyocytes (Krishnan et al., 2009) through activation of the HIF-1 pathway. Therefore, it could be argued that lipid accumulation in myopathic muscles could be caused in part by the upregulation of *PPARγ* by the HIF-1 pathway in these muscles but further research is required to confirm this in broiler breast muscles. Furthermore, the upregulation of the HIF-1 pathway can enhance lipogenesis by increasing the expression of fatty acid binding proteins or FABPs (Mylonis et al., 2019). The expression of genes encoding FABP3 and FABP4 was also shown to be upregulated in WB-affected muscles (Lake et al., 2019). FABPs are a family of proteins that act as lipid chaperones, actively facilitating lipid transport to specific compartments of the cell and their storage in lipid droplets (Furuhashi and Hotamisligil, 2008). Mice deficient in FABP3 exhibited severely inhibited uptake of long-chain fatty acids in the heart and skeletal muscles (Binas et al., 1999). An upregulation of this gene through HIF-1α could thus contribute to lipid accumulation in myopathic muscles.

**FIGURE 6:**
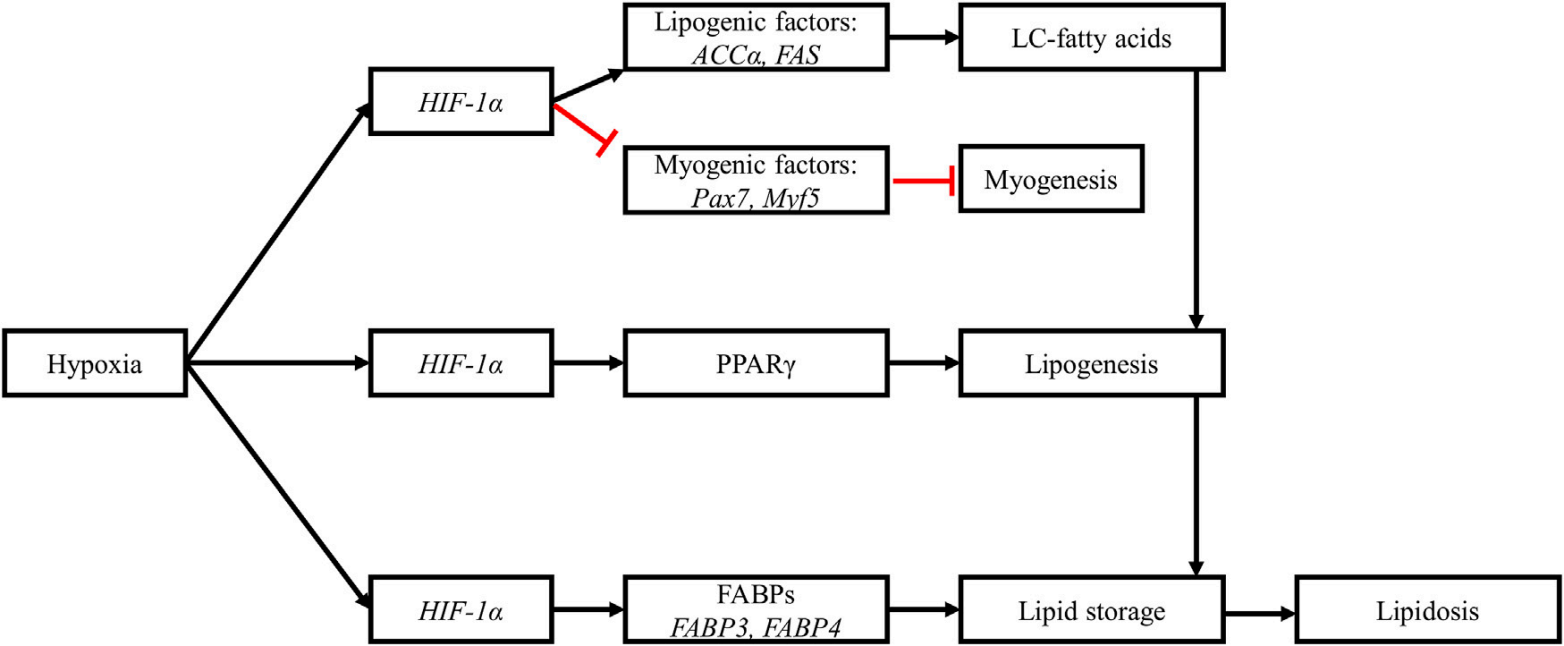
Hypoxia alters muscle lipid metabolism. Upregulation of *HIF-1α* downregulates myogenic factors while upregulating lipogenic factors. The increased expression of acetyl-CoA carboxylase *a* (*ACCα*) and fatty acid synthase (*FAS*) promotes the synthesis of long-chain fatty acids in myopathic muscles. The upregulation of *HIF-1α* is also associated with increased expression of peroxisome proliferator-activated receptor gamma (*PPARγ*), leading to increased adipogenesis and accumulation of lipids in myopathic muscles. Hypoxia also upregulates the expression of fatty acid binding proteins (FABPs), further stimulating lipogenesis and lipid storage in muscle tissues. These hypoxia-induced changes are responsible for the observed lipidosis in myopathic muscles. Black arrows: upregulation, blunt red arrows: downregulation.

To summarize (Figure 6), through the activation of the HIF-1 pathway, hypoxia upregulates pathways involved in fatty acid synthesis, lipogenesis and in lipid storage, which results in pathological lipid accumulation in myopathic breast muscles.

### 5.5 Hypoxia and fibrosis in myopathic muscles

As discussed earlier, BMM are characterized by the necrosis and degeneration of muscle fibers and the infiltration of the damaged muscle tissue by inflammatory cells. The degeneration and inflammation of the *P. major* muscle induce an increased deposition of extracellular matrix (ECM) proteins, leading to fibrosis, which is a microscopic characteristic of WB and WS (Kuttappan et al., 2013; Soglia et al., 2016a; Velleman, 2020). In a study that used proteomic data from WB-affected muscles of 52-day-old broiler chickens, an analysis of the upstream regulators of this myopathy showed that the transforming growth factor β1 (TGF-β1) was activated (Bottje et al., 2021). This cytokine is one of three members of TGF-β family, which is involved in a wide range of cellular events and is known to promote fibrosis in skeletal muscles (Delaney et al., 2017). TGF-β1 produces its cellular effects by binding to its receptor TβRII. This dimer then phosphorylates the TβRI receptor. This phosphorylated receptor in turn phosphorylates SMAD2 and SMAD3, two members of the SMAD signaling pathway (Schmierer and Hill, 2007). Consequently, the activated SMAD2 and SMAD3 form a complex with SMAD4, which promotes the translocation of the complex to the nucleus to induce the expression of their target genes, including genes that encode proteins of the ECM such as fibronectin and collagen (Delaney et al., 2017). A recent study investigated the mechanisms underlying the fibrosis of WB-affected muscles sampled from commercial broilers at 6 weeks of age (Xing et al., 2021). These authors showed that the expression of all three members of the TGF-β family was significantly increased in WB-affected muscles compared with unaffected muscles at both the mRNA and protein levels. Consequently, the expression of fibronectin and collagen was also significantly increased in WB-affected muscles at the mRNA and protein levels. The deposition of large amounts of connective tissue fibers in a parallel and highly packed fashion in the interstitial spaces gives WB-affected muscles their characteristic hardness (Velleman, 2020).

**FIGURE 7:**
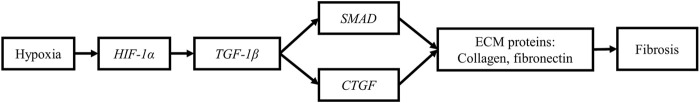
Hypoxia promotes fibrosis in myopathic muscles. The upregulation of *HIF-1α* promotes the synthesis and deposition of extracellular matrix (ECM) proteins (collagen and fibronectin) in myopathic muscles through its interaction with the transforming growth factor β1/SMAD signaling pathway and its upregulation of connective tissue growth factor (CTGF).

Hypoxia has been recently shown to increase the expression of collagen genes (*Col1A1* and *Col1A2*) and proteins (Col1) in chicken primary myoblasts contributing to fibrosis (Greene et al., 2023). Hypoxia can contribute to fibrosis in two ways (Figure 7). It has been shown to activate the TGF-β1/SMAD signaling pathway through HIF-1α signaling, leading to increased collagen deposition in human epidermal fibroblasts (Mingyuan et al., 2018). Furthermore, HIF-1α signaling act synergistically with TGF-β1 to increase the expression of the connective tissue growth factor (CTGF) in myotubes of different models of skeletal muscles (Valle-Tenney et al., 2020). CTGF is a potent profibrotic growth factor that increases the expression of the ECM components, thus contributing to fibrosis (Vial et al., 2008). The expression of this growth factor was significantly higher in WB-affected than in unaffected muscles (Xing et al., 2021). Thus, it can be argued that under hypoxic conditions in myopathic muscles, HIF-1α can interact with TGF-β1, leading to increased expression of CTGF, which in turn increases secretion of the ECM components including fibronectin and collagen. The increased deposition of these components in the *perimysium* and *endomysium* produces the form of fibrosis that characterizes myopathic breast muscles. Further research is needed to better understand the pathways by which hypoxia contributes to fibrosis in broiler breast muscle.

## 6 Hypoxia alters mitochondrial morphology, dynamics, and functions in myopathic muscles

In muscles composed of type IIB myofibers, such as *P. major*, mitochondria have a small size (0.15 vs. 0.35 µm^2^ in type I), an ellipsoid shape, and are sparsely distributed among myofibrils (Hosotani et al., 2021). According to these authors, individual mitochondria from type IIB myofibers exhibited fewer interconnections and formed less extensive networks than mitochondria from type I myofibers. With age, mitochondria in the *P. major* muscle shift their position from the center of myofibers to the zone adjacent to blood capillaries as an adaptation to decreased blood flow and increased diffusion distances which facilitates access to oxygen (Belichenko et al., 2004).

In myopathic muscles, mitochondria exhibit hyperplasia and they aggregate in large numbers (up to 40 mitochondria) between myofibrils (Sihvo et al., 2018). These mitochondria also exhibit morphological changes including vacuolation, swollen matrix, loss of cristae, and irregular shape (Sihvo et al., 2018; Hosotani et al., 2020; Pan et al., 2021). These studies suggested that changes in mitochondrial morphology were associated with the hypoxic state of myopathic muscles.

### 6.1 Hypoxia stimulates mitochondrial fission and fusion in myopathic muscles

Mitochondria are highly dynamic organelles that undergo fission (*i.e.,* division of one mitochondrion into two daughter mitochondria) and fusion (*i.e.,* one mitochondrion produced by a fusion of two mitochondria) to maintain their shape, size, and cellular distribution (Tilokani et al., 2018; Chen et al., 2022). These processes are fundamental in allowing mitochondria to respond to cellular stress conditions and to help cells adapt to such conditions (Wang et al., 2022).

Hypoxia, through the activation of the HIF-1 pathway, has been associated with changes in the expression of genes regulating mitochondrial dynamics to protect mitochondria from oxidative stress (Li et al., 2019). In a recent study of WB-affected muscles sampled from 50-day-old commercial broilers, the expression of *HIF-1α* was positively correlated (*r* = 0.33, *p* = 0.049) with dynamin-related protein 1 (*DRP1*), a gene involved in mitochondrial fission (Hosotani et al., 2020). The promotion of mitochondrial fission by upregulating *DRP1* helps segregate mitochondria impaired by hypoxia-induced cellular stress. This enables their selective elimination to maintain mitochondrial function which is essential for cell survival (Li et al., 2019; Wang et al., 2022).

Mitochondrial fusion also helps in alleviating stress because it combines the contents of partially damaged mitochondria, which limits excessive mitochondrial clearance (Youle and van der Bliek, 2012). This process is regulated by genes including mitofusion (*MFN*) 1 and 2 (Tilokani et al., 2018). Proteins encoded by *MFN1* and *MFN2* maintain mitochondrial networks by binding mitochondria together and initiating the fusion of their outer membrane (Lugus et al., 2011). In WB-affected muscles, the expression of *HIF-1α* was not directly correlated with that of *MFN1* or *MFN2*; however, *HIF-1α* upregulation induced an upregulation of the *VEGF-A* gene encoding the vascular endothelial growth factor A (Hosotani et al., 2020), an essential mediator of skeletal muscle angiogenesis and a well-known transcriptional target of HIF-1 (Rodriguez-Miguelez et al., 2015). The upregulation of *VEGF-A* also upregulates *MFN1* and *MFN2* (Lugus et al., 2011; Rodriguez-Miguelez et al., 2015). This relationship was observed in WB-affected muscles, where the expression of *VEGF-A* was significantly correlated with that of *MFN1* (r = 0.68, *p* < 0.0001) and *MFN2* (r = 0.75, *p* < 0.0001) (Hosotani et al., 2020) suggesting an indirect stimulation of mitochondrial fusion by HIF-1.

Therefore, the upregulation of the HIF-1 pathway in myopathic muscles seems to directly stimulate mitochondrial fission and indirectly stimulate mitochondrial fusion. Both mechanisms reduce cellular stress while eliminating defective mitochondria to reduce the risk of muscle tissue damage or to limit further progression of myopathies. However, under persistent cellular stress, these mechanisms could be overwhelmed, leading to the above-described changes in mitochondrial morphology. The subsequent mitochondrial damage can also contribute to the dysregulated energy metabolism in myopathic muscles (see Section 4.4 and Section 5.3).

### 6.2 Hypoxia induces an oxidative stress in myopathic muscles

The mitochondrial respiratory chain is a major source of reactive oxygen species or ROS (Murphy, 2009). These molecules are produced during muscle contraction and their role depends on their concentration and on the duration of cellular components exposure to these molecules. Under high concentrations and long exposure times, ROS pose significant danger to mitochondria and other cellular components. Under low concentrations and short exposure times, ROS are signaling molecules that regulate multiple physiological processes in skeletal muscles (Barbieri and Sestili, 2012). In broiler breast muscles, superoxide, a primary ROS, is formed at complex I and complex III of the, ETC (Hakamata et al., 2020). Superoxide is formed when electrons leak from these complexes and are transferred to molecular oxygen (Murphy, 2009). Excessive amounts of superoxide undergo dismutation by superoxide dismutase (SOD), generating hydrogen peroxide (Wang et al., 2018), which is another member of the ROS family. Hydrogen peroxide is then converted to water and oxygen by glutathione peroxidase or catalase (Kozakowska et al., 2015).

The mitochondrial damage reported in myopathic muscles, coupled with the metabolic shift toward further anaerobic glycolysis, can lead to decreased mitochondrial respiratory activity, highlighted by lower citrate synthase activity (Pan et al., 2021; Li et al., 2022) and lower mitochondrial membrane potential reported in myopathic muscles (Pan et al., 2021). As the respiratory chain is slowed, the likelihood of electron leakage and transfer to molecular oxygen increases, which can increase the production of superoxide (Thomas and Ashcroft, 2019). According to the phenomenon of ROS-induced ROS release, the production of a small amount of ROS by a small number of mitochondria can propagate to other mitochondria via the mitochondrial network, leading to increased mitochondrial ROS production and eventually promote ROS production from non-mitochondrial sources (Park et al., 2011). This could partly explain the significantly higher levels of ROS reported in WB-affected (+15%, *p* < 0.01) muscles (Pan et al., 2021) and in WS-affected (+57%, *p* < 0.001) muscles (Salles et al., 2019) compared with unaffected muscles.

The elevated levels of ROS induce a response from the antioxidant defense system in myopathic muscles. Compared with unaffected muscles, muscles moderately affected by WS exhibited a significant increase in the activity of SOD, glutathione peroxidase, and catalase (Salles et al., 2019; Carvalho et al., 2021). Under persistent oxidative stress, the antioxidant defense system becomes overwhelmed by oxidative damage coupled with depletion of antioxidant molecules (Abasht et al., 2016), resulting in the decreased activity of the antioxidant enzymes in muscles severely affected by this myopathy (Salles et al., 2019; Carvalho et al., 2021). A similar response of antioxidant enzymes has also been reported in WB-affected muscles (Praud et al., 2020; Pan et al., 2021). The response of the antioxidant defense system to increased ROS production in myopathic muscles is partly mediated by the nuclear factor erythroid 2-related factor 2 (Nrf2) pathway (Pan et al., 2021). Nrf2, a master transcriptional regulator responsible for cellular redox hemostasis, is sequestered in the cytoplasm by Kelch-like ECH-associated protein 1 (Keap1) and is rapidly degraded by the Cullin 3 ubiquitin–proteasomal system under physiological conditions (Surai et al., 2019; Gao et al., 2021). Under oxidative stress conditions, Nrf2 dissociates from Keap1 and is translocated to the nucleus (Bellezza et al., 2018). In the nucleus, Nrf2 binds either directly to antioxidant response elements (ARE) or indirectly to ARE-like sequences within the Nrf2 promotor, which leads to the upregulation of genes involved in the antioxidant defense system, including genes encoding SOD, catalase, and glutathione peroxidase (Gao et al., 2021). Hypoxia, through the upregulation of *HIF-1α*, upregulates Nrf2 expression at the protein level and its binding to ARE leading to an upregulation of genes encoding antioxidant enzymes in mouse skeletal muscles (Ji et al., 2018). Therefore, the upregulation of the Nrf2 pathway and the subsequent upregulation of genes encoding for SOD and glutathione peroxidase evidenced in WB-affected muscles (Pan et al., 2021) can be attributed to hypoxia and to the hypoxia-induced oxidative stress in these muscles.

### 6.3 Hypoxia-induced oxidative damage in myopathic muscles

The increased production of ROS in myopathic muscles due to hypoxia results in a concomitant increase of lipid peroxidation, as well as protein and DNA oxidation products. The thiobarbituric acid–reactive substances (TBA-RS) index is a widely used method to assay secondary products of lipid peroxidation (Sammari et al., 2023). Using this method, multiple studies have reported significantly increased levels of malondialdehyde (MDA) in WS- (Carvalho et al., 2021; Pereira et al., 2022) and WB-affected muscles, indicating elevated levels of lipid peroxidation (Soglia et al., 2019; Pan et al., 2021; Li et al., 2022). Carbonyl and thiol groups in muscle and meat are widely used markers of protein oxidation (Soglia et al., 2016b). In myopathic muscles, proteins are exposed not only to ROS but also to other pro-oxidant molecules, such as lipid peroxidation products. Consequently, proteins in these muscles undergo oxidation as seen through their significantly elevated levels of carbonyl and thiol groups (Carvalho et al., 2021; Li et al., 2022). Other markers of lipid peroxidation and protein oxidative damage have also been reported in the literature. For instance, eicosanoids, including 15-HETE and 15-KETE (both metabolites of arachidonic acid), were shown to be among top metabolites differentiating WB-affected from unaffected muscles (Abasht et al., 2016). In this same study, 1-methylhistidine, an indicator of skeletal muscle oxidative stress, and 3-methylhistidine, a known marker of protein breakdown, were also shown to be present in higher quantities in WB-affected than unaffected muscles.

**FIGURE 8:**
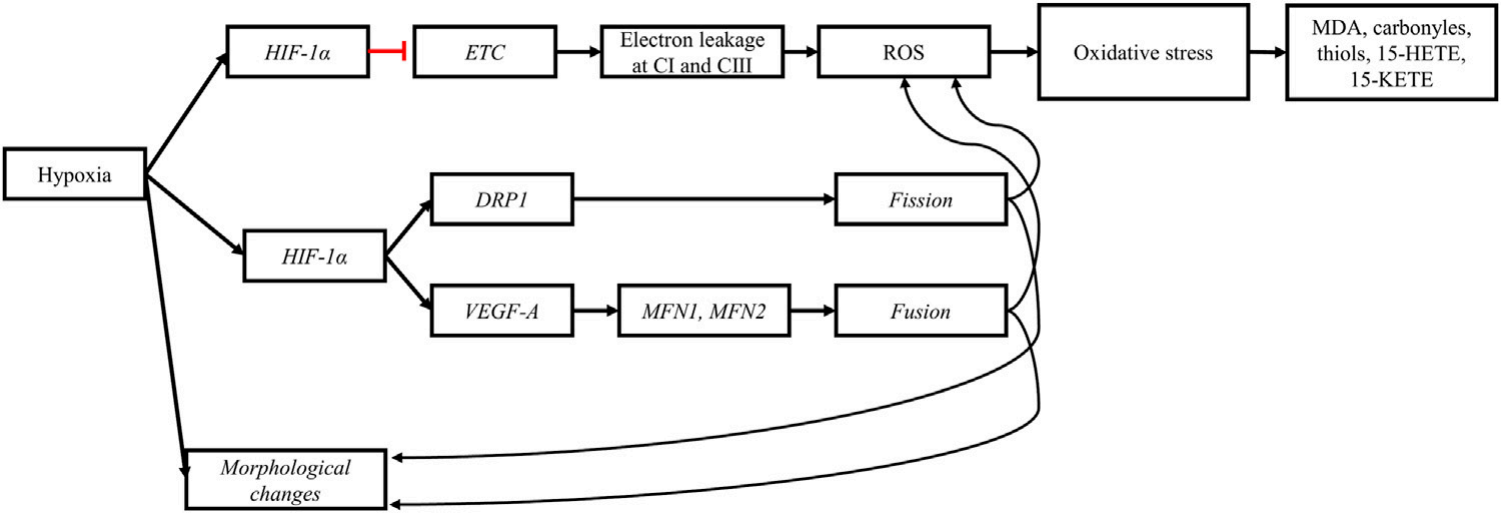
Hypoxia impairs mitochondrial morphology and functions. The perturbation of the tricarboxylic acid cycle (TCA) induced by the upregulation of *HIF-1α* slows the electron transport chain (ETC.), increasing the probability of electron leakage at complex I (CI) and III (CIII). The leaked electrons bind to molecular oxygen to form reactive oxygen species (ROS). High concentrations of ROS and prolonged exposure of cellular components to ROS lead to oxidative stress and subsequently to lipid peroxidation and protein oxidation. The products from oxidative damage (MDA, carbonyls, thiols, 15-HETE, and 15-KETE) accumulate in myopathic muscles. Furthermore, the upregulation of *HIF-1α* upregulates genes responsible for mitochondrial fission (*DRP1*) and fusion (*VEGF-A*, *MFN1*, and *MFN2*). The stimulation of mitochondrial dynamics is a response mechanism aiming to alleviate cellular stress and eliminate defective mitochondria induced by hypoxia. Black arrows: upregulation, blunt red arrows: downregulation.

In summary (Figure 8), under hypoxic conditions, lipid accumulation increases in myopathic muscles, and energy metabolism is shifted away from the TCA cycle to reduce oxygen consumption. These changes, coupled with altered mitochondrial functions, lead to increased ROS production. When the antioxidant defense system becomes overwhelmed by persistent ROS exposure, oxidative damage develops. This is often manifested by elevated levels of products and markers of lipid peroxidation and protein oxidation. Hypoxia-induced oxidative stress can therefore contribute to the initiation and progression of the pathological changes underlying BMM.

## 7 Hypoxia-induced apoptosis in myopathic muscles

Breast muscle growth increases with age leading to increased muscle fiber diameter and decreased myofiber area to blood capillary (Sihvo et al., 2018). These changes in muscle histology are associated with increased myofiber necrosis and degeneration due to ischemia and hypoxia (Joiner et al., 2014). Radaelli et al. (2017) evaluated muscle fiber degeneration in the *P. major* muscle of commercial strains of broiler chickens and showed that the percentage of myofibers with signs of degeneration increased from 28.1% at 21 days to 96.6% at 46 days while the number of nuclei showing signs of apoptotic processes increased from 0.44 at 21 days to 8.45 per examined field at 46 days. Interestingly, increase in the apoptotic and degenerative processes in the *P. major* muscle reported in this study coincided with the previously reported increase in the expression of *HIF-1α* in this muscle from 21 to 42 days (Young and Rasmussen, 2020; Malila et al., 2022) which provided further support for the key role of hypoxia in triggering the histopathological changes that characterize myopathic muscles. In skeletal muscles, caspases are one of the major regulators of apoptotic signaling (Quadrilatero et al., 2011), and they are activated under hypoxic conditions. In a recent study, Greene et al. (2023) assessed the mRNA levels of genes encoding for caspase-1, cappase-3 and caspase-9 in chicken primary myoblasts culture that was exposed to hypoxia for 0, 8 and 24 h. The mRNA level of genes encoding all three proteases increased significantly after 24 h under hypoxic conditions (1% O_2_, 5% CO_2_ and 94% N_2_). These authors further showed that the protein level of caspase-3 evaluated by Western blotting was significantly increased after 24 h under hypoxic conditions. Caspase-9 is an initiator caspase that promotes the downstream activation of caspase-3 which is an executioner or effector caspase responsible for the ultimate degradation of cellular content (Sendoel and Hengartner, 2014). Damage to DNA and stress of the sarcoplasmic reticulum, which both have been reported in myopathic muscles (Radaelli et al., 2017; Greene et al., 2023), are considered two of the most important signaling pathways upregulating the apoptotic activity of caspases (Quadrilatero et al., 2011). Once activated, effector caspases dismantle cellular components that could induce inflammation to prevent further damage to muscle tissue (Fink and Cookson, 2005). However, the specific role of the caspase signaling pathway in the development of BMM phenotypes requires further research.

**FIGURE 9:**
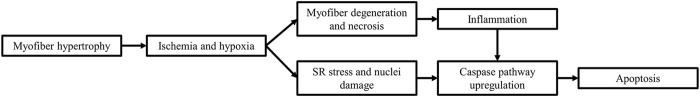
Hypoxia and ischemia stimulate apoptosis in myopathic muscles. The ischemic and hypoxic state of myopathic muscle is responsible for necrosis and myofiber degeneration, inducing an inflammatory response in affected muscles. Moreover, hypoxia stresses the sarcoplasmic reticulum (SR) and damages the nuclei of affected muscle tissue. The inflammation, SR stress, and nuclei damage upregulate the caspase signaling pathway, ultimately dismantling cellular proteins, and leading to apoptosis as a response mechanism to limit further damage to muscle tissue.

To summarize (Figure 9), the damaged nuclei and stressed sarcoplasmic reticulum associated with hypoxia in myopathic muscles activate the caspase pathway leading to apoptosis in response to myofiber necrosis.

## 8 The big picture

The continually increasing worldwide demand for poultry meat necessitates the development of broiler strains that allow the industry to meet the increasing demand while reducing the effect of broiler production on the environment.

**FIGURE 10:**
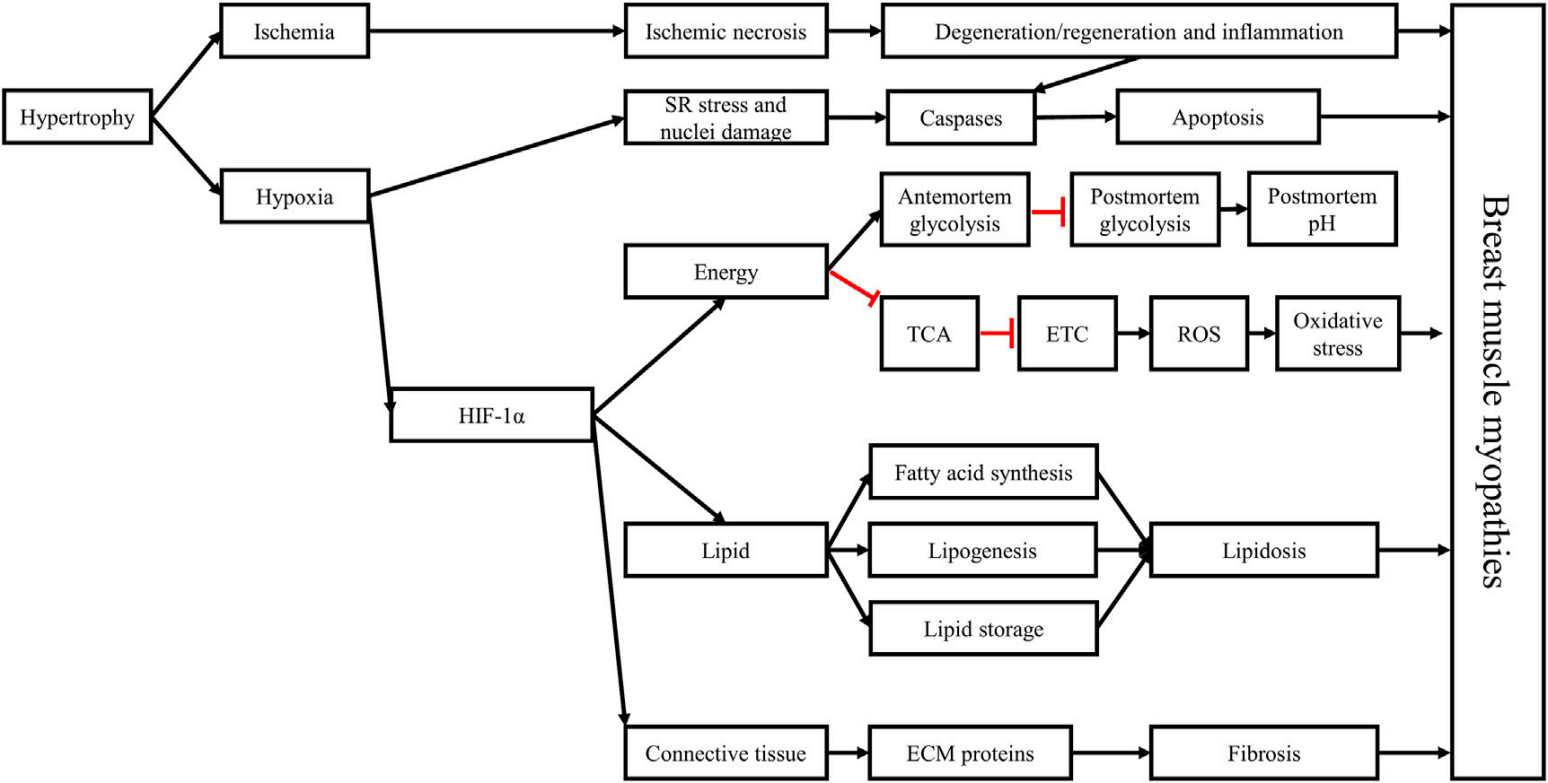
Hypoxia plays a critical role in the development of breast muscle myopathies through the activation of the hypoxia-inducible factor 1 (HIF-1) pathway. The hypertrophic growth of breast muscles is associated with decreased capillary density, leading to decreased circulation (ischemia) and oxygenation (hypoxia) in muscle tissue, coupled with lower nutrient supply. The histopathological changes associated with breast muscle myopathies, including necrosis, myofiber degeneration, and inflammation, are consequences of the ischemic and hypoxic states. Hypoxia can stress the sarcoplasmic reticulum and damage the nuclei, leading to the activation of the caspase signaling pathway, triggering apoptosis. Moreover, HIF-1α can further stimulate antemortem anaerobic glycolysis in the *Pectoralis major* muscle, depleting glycogen reserves by the time birds reach market age, contributing to the early cessation of *post-mortem* acidification in this muscle which explain the higher ultimate pH in these muscles. Furthermore, HIF-1α slows the TCA cycle and impedes mitochondrial function, leading to increased ROS production and accumulation, triggering a state of oxidative stress in the muscle and damaging its cellular components. HIF-1α also alters lipid metabolism in the *Pectoralis major* muscle, leading to increased synthesis of long-chain fatty acids, lipogenesis, and lipid storage, ultimately causing lipidosis. Finally, by interacting with the TGF-β1/SMAD pathway and with the connective tissue growth factor, HIF-1α increases the deposition of extracellular matrix (ECM) proteins in muscle tissue, leading to fibrosis. These structural and metabolic changes trigger the onset and development of breast muscle myopathies and give them their distinctive phenotypes. Black arrows: upregulation, blunt red arrows: downregulation.

Genetic selection is one of the most important tools used by the industry to create and develop fast-growing and high-yielding strains of broiler chickens. The increased selection pressure to continuously improve broiler BMY leads to increased muscle fiber hypertrophy in the pectorals of these birds, reducing the capillary density of these muscles, and inducing an ischemic and hypoxic states. Hypoxia activates and upregulates the HIF-1 pathway (Figure 10). This transcription factor upregulates the expression of numerous hypoxia-responsive genes, leading to an upregulation of anaerobic glycolysis and lipogenesis, a downregulation of the TCA cycle, a slowdown of the, ETC, and an increase of pro-oxidant molecules coupled with a decrease of antioxidant molecules. The pro-oxidant molecules target lipids and proteins in the cell membrane and in cellular organelles, leading to their oxidation and the further release of pro-oxidant molecules, inducing further oxidative damage and overwhelming the antioxidant defense system.

Ischemia and hypoxia-induced oxidative stress can then initiate the multiphasic degenerative process in muscle tissue. In response to necrosis and inflammation, satellite cells are activated to repair the necrotic damage once inflammatory cells infiltrate the damaged muscle tissue and clean the zone from necrotic debris. Under the persistent hypoxic state, the caspase signaling pathway is upregulated initiating apoptosis as an attempt to control inflammation. In addition, satellite cells change their program and instead of differentiating to myoblasts to form new myotubes that replace damaged muscle fibers, they become lipogenic, resulting in the increased synthesis of fatty acids and fat deposition in muscle tissue. Simultaneously, hypoxia acts synergistically with the transforming growth factor β1 to upregulate the connective tissue growth factor, which in turn inhibits myogenesis and increases the expression of the extracellular matrix proteins, leading to fibrosis. Lipidosis and fibrosis replace muscle tissue with fat and connective tissue, resulting in the distinctive phenotypic characteristics of BMM.

## 9 Conclusions and perspectives

Hypoxic and oxidative damages resulting from the reduced capillary density in the *P. major* muscle are critical determinants of the onset and progression of BMM. Although an important progress has been achieved on understanding the role of hypoxia in the development of BMM, further research is needed to better understand the relationship between the HIF-1 pathway and other pathways involved in the occurrence of BMM. In particular, the interaction between HIF-1 and the TGF-β family requires further investigation in relation with fibrosis and the development of the specific and distinct phenotypes of BMM. More research is also required to confirm the relationship between HIF-1 and PPARγ and the consequences of this relationship for pathological lipid storage in muscle tissue. In addition, research is needed to advance our understanding of the role of the caspase signaling pathway in the development of BMM under hypoxic stress. In terms of solutions for BMM, future research should focus on developing strategies to improve both the vasculature and the antioxidant status of breast muscles to limit the occurrence and severity of these myopathies. In the past, developmental problems caused by genetic selection were mostly resolved by genetic selection, with leg deformities and ascites being prime examples of the role of genetic selection in resolving genetic issues. Given that BMM are moderately to highly determined by genetics, genetic selection would be highly efficient in reducing their occurrence or at least their severity. However, given the genetic and phenotypic positive association between BMM and increased BMY, selecting directly against these myopathies could reduce BMY, which is undesirable. Consequently, research is needed to identify new selection criteria in relation to muscle structure (*e.g.*, capillary density, ratio of muscle fiber number to blood capillaries, width of interstitial spaces, density and organization of connective tissue) and in relation to muscle content of naturally occurring antioxidant molecules (*e.g.*, histidine-containing dipeptides including carnosine and anserine and their derivatives). Research is also needed to test the feasibility of selection for such criteria and the efficacy of the selection process in reducing the occurrence and severity of BMM with limited impact on BMY. However, because of the pyramidal organization of broiler breeding schemes, results from this approach would not be available in the short term; therefore, other approaches need to be developed in parallel to the genetic selection-based approach. Embryonic manipulations (*e.g.*, acclimatization to lower oxygen levels during the critical phase of development of the pectoral muscles) and the use of *in ovo* feeding with substances that upregulate the expression of angiogenic genes during embryogenesis could be explored. Nutritional strategies to improve the antioxidant status of breast muscle using naturally occurring antioxidants (*e.g.*, carnosine) should also be further explored. Finally, the reduction of the occurrence and severity of BMM with limited undesirable consequences for economically important traits would probably require a combination of genetic selection with improved nutritional and managerial strategies.
